# Supplementary material for: De novo design of protein nanoparticles with integrated functional motifs
Source: bioRxiv. 2026 Jan 2:2025.12.19.695620. Preprint. [Version 2] doi: 10.64898/2025.12.19.695620 (PMC12776285; doi:10.64898/2025.12.19.695620)
Supplement: Supplement 1 [file NIHPP2025.12.19.695620v2-supplement-1.pdf]

bioRxiv preprint doi: https://doi.org/10.64898/2025.12.19.695620; this version posted January 2, 2026. The copyright holder for this preprint (which was not certified by peer review) is the author/funder, who has granted bioRxiv a license to display the preprint in perpetuity. It is made available under aCC-BY 4.0 International license.

Supplementary Data

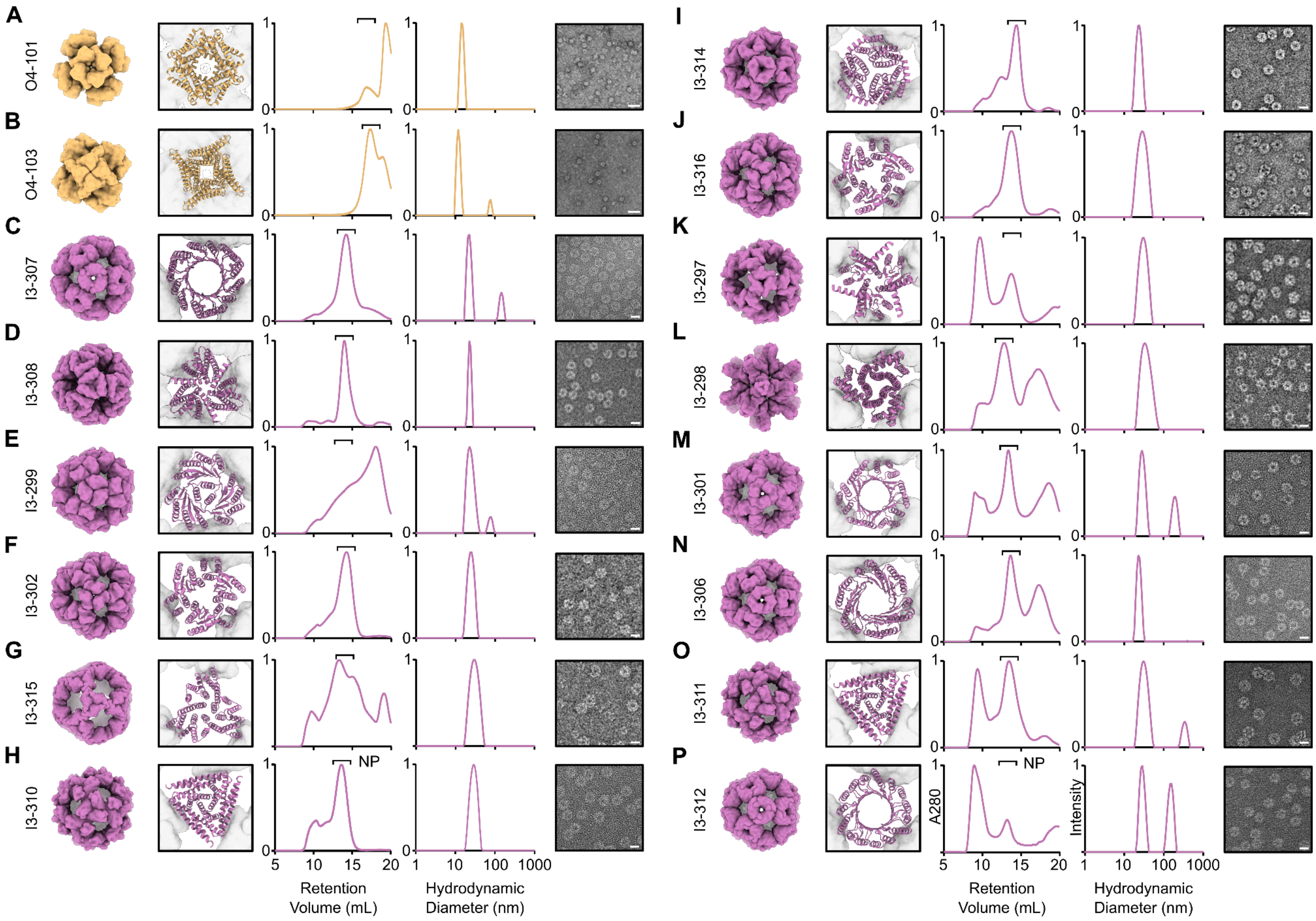

Figure S1. Characterization of de novo nanoparticles generated with unconditional diffusion of components. *From left to right:* Design models, SEC, DLS (intensity), and negatively stained electron micrographs for (**A**) O4-101, (**B**) O4-103, (**C**) I3-307, (**D**) I3-308, (**E**) I3-299, (**F**) I3-302, (**G**) I3-315, (**H**) I3-310, (**I**) I3-314, (**J**) I3-316, (**K**) I3-297, (**L**) I3-298, (**M**) I3-301, (**N**) I3-306, (**O**) I3-311, and (**P**) I3-312. The design model of the nanoparticle is shown at left and a cropped image of the design model for the diffused oligomer in the context of the nanoparticle is shown at right. nsEM scale bars = 20 nm.

bioRxiv preprint doi: https://doi.org/10.64898/2025.12.19.695620; this version posted January 2, 2026. The copyright holder for this preprint (which was not certified by peer review) is the author/funder, who has granted bioRxiv a license to display the preprint in perpetuity. It is made available under aCC-BY 4.0 International license.

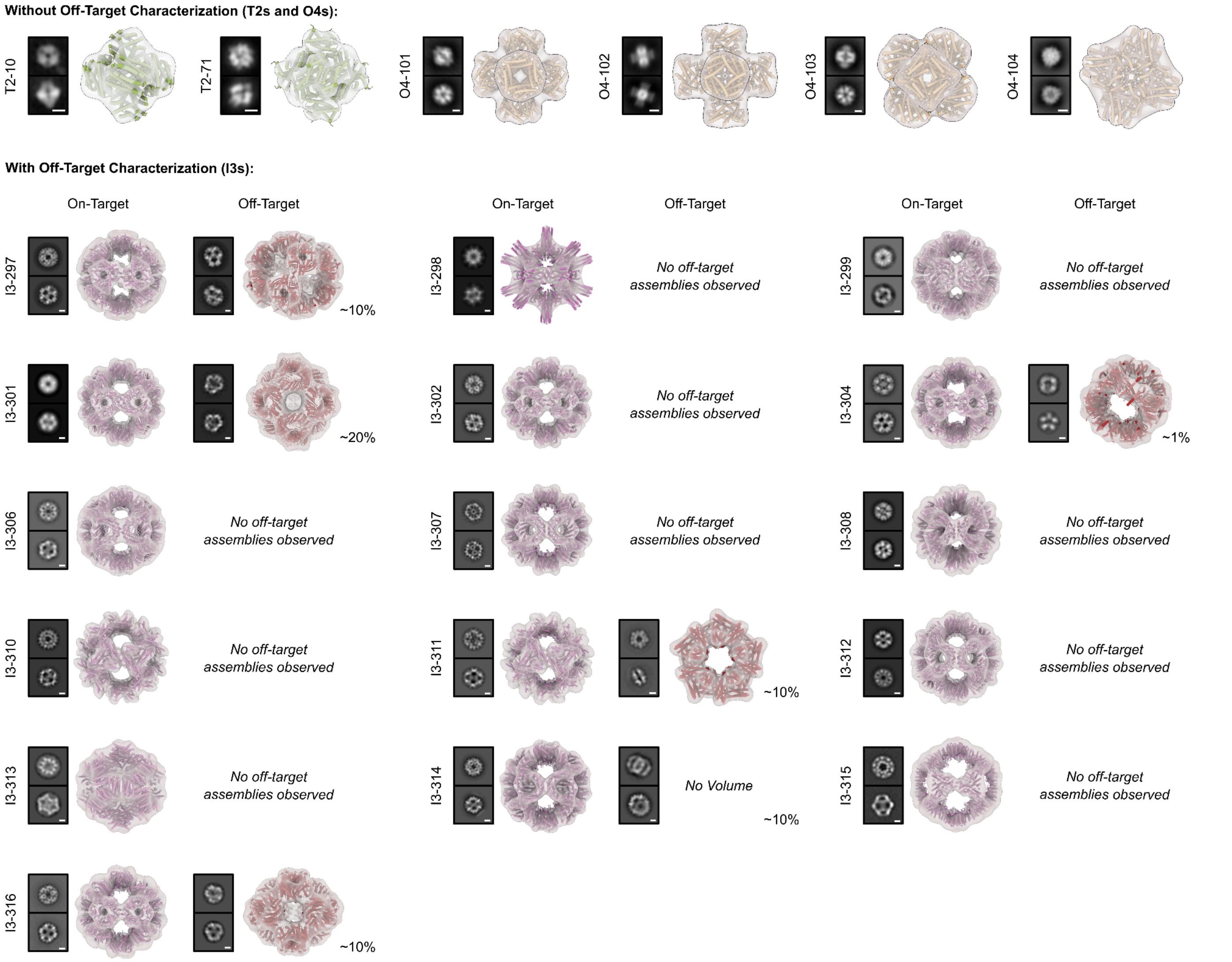

Figure S2. nsEM analysis of unconditionally diffused nanoparticles.
Left, 2D class averages and *right,* 3D reconstructions of on-target nanoparticles and off-target assemblies (when present). Off-target species were only analyzed for successfully designed icosahedral nanoparticles. Reconstructions show the map in transparent grey with the model of the protein nanoparticle shown underneath as a cartoon in pink (icosahedral), yellow (octahedral), green (tetrahedral), or red (off-target). Relative abundances of observed off-target species are approximated next to each species. Class averages for nanoparticles shown in Figure 2 are replicated here for clarity. Scale bars = 5 nm.

bioRxiv preprint doi: https://doi.org/10.64898/2025.12.19.695620; this version posted January 2, 2026. The copyright holder for this preprint (which was not certified by peer review) is the author/funder, who has granted bioRxiv a license to display the preprint in perpetuity. It is made available under aCC-BY 4.0 International license.

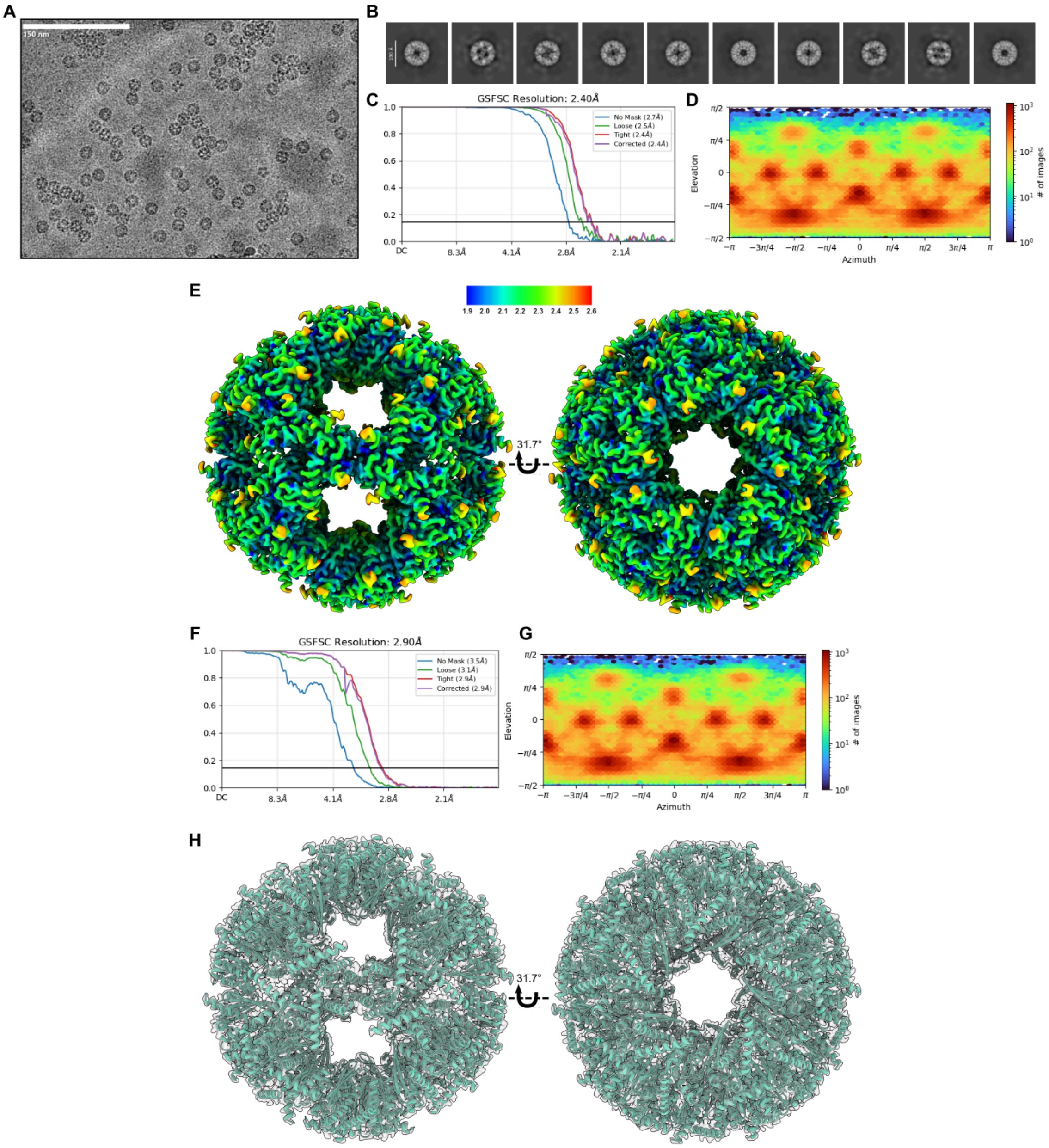

Figure S3. Cryo-EM analysis of de novo designed octahedral nanoparticle I3-304.
(**A**) Representative cryo-EM micrograph. (**B**) Representative 2D class averages of the dominant on-target I3-304 nanoparticle. (**C**) Global gold-standard FSC from non-uniform refinement with icosahedral symmetry applied, yielding a resolution of 2.90 Å at the 0.143 cutoff. (**D**) Orientational distribution plot demonstrating full angular sampling. (**E**) Local resolution map (0.143 FSC cutoff). (**F**) Global gold-standard FSC from non-uniform refinement with C1 symmetry applied. (**G**) Orientational distribution plot from C1 refinement. (**H**) Icosahedral atomic model docked into the C1 map, showing no detectable deviations from imposed symmetry.

bioRxiv preprint doi: https://doi.org/10.64898/2025.12.19.695620; this version posted January 2, 2026. The copyright holder for this preprint (which was not certified by peer review) is the author/funder, who has granted bioRxiv a license to display the preprint in perpetuity. It is made available under aCC-BY 4.0 International license.

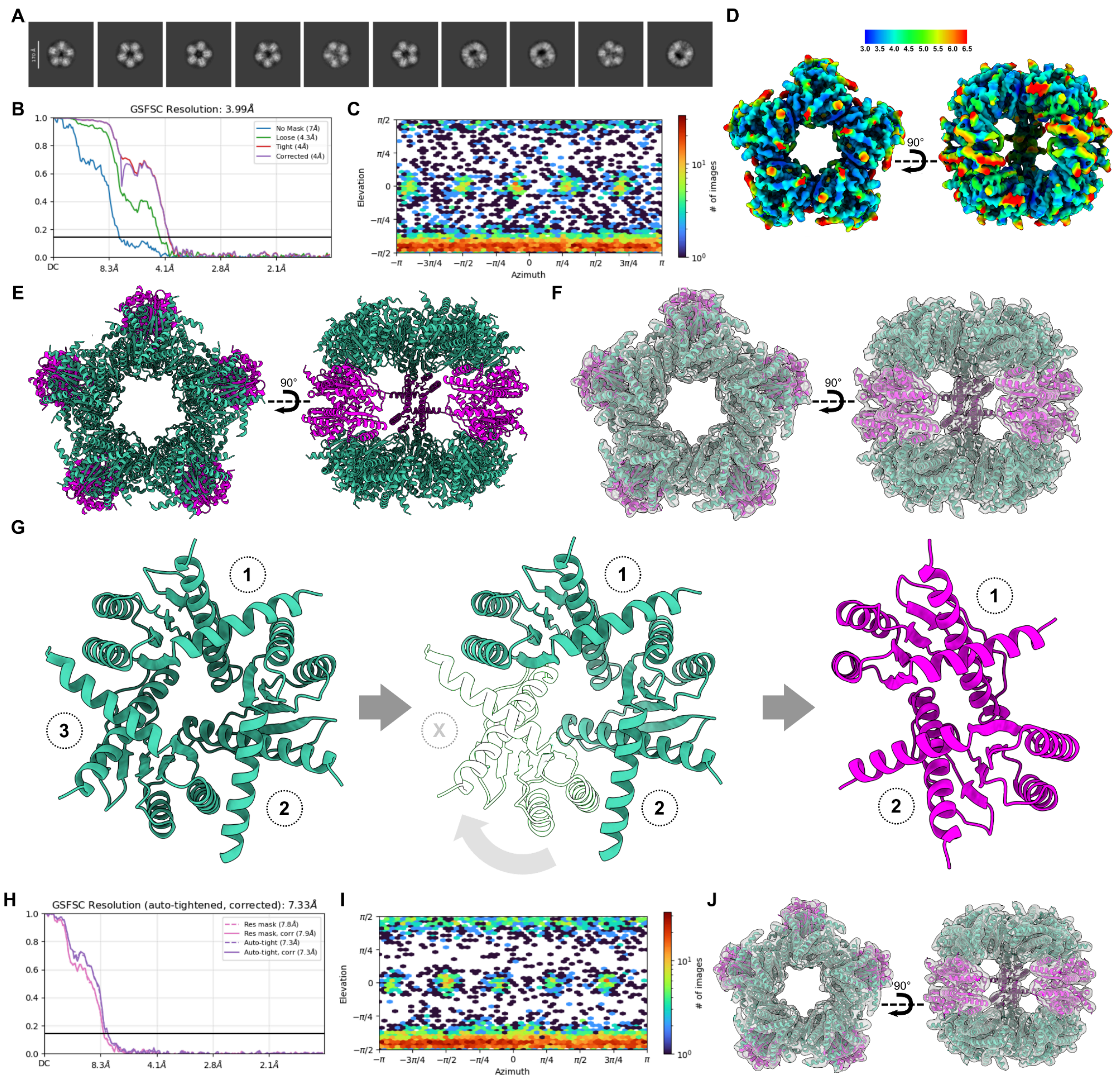

Figure S4. Cryo-EM analysis of the I3-304 off-target D5 nanoparticle assembly.
(**A**) Representative 2D class averages. (**B**) Global gold-standard Fourier shell correlation (GSFSC) from non-uniform refinement with D5 symmetry, yielding a resolution of 3.99 Å at the 0.143 cutoff. (**C**) Orientational distribution plot. (**D**) Local resolution map (0.143 FSC cutoff). (**E**) Atomic model of the off-target D5 assembly. The assembly comprises 40 subunits, arranged as two sets of five trimers (green) connected by five off-target dimers (pink). (**F**) Atomic model docked into the 3.99 Å D5-symmetrized cryo-EM density map, demonstrating high model-to-map agreement. (**G**) Structural analysis of the off-target dimer observed at the dihedral two-fold axes. *Left,* the on-target trimeric building block; *middle,* elimination of subunit X of the trimer and rotation of subunit 2 leads to *right,* the off-target dimer (pink), which forms a C2-symmetric dimer in the absence of the third chain. (**H**) Global gold-standard Fourier shell correlation (GSFSC) from non-uniform refinement performed in C1 symmetry, yielding a resolution of 7.33 Å at the 0.143 cutoff. (**I**) Orientational distribution plot for the C1 refinement. (**J**) D5 atomic model rigid-body docked into the C1 cryo-EM density map, showing no evidence of symmetry deviations and confirming the D5 architecture of the off-target assembly.

bioRxiv preprint doi: https://doi.org/10.64898/2025.12.19.695620; this version posted January 2, 2026. The copyright holder for this preprint (which was not certified by peer review) is the author/funder, who has granted bioRxiv a license to display the preprint in perpetuity. It is made available under aCC-BY 4.0 International license.

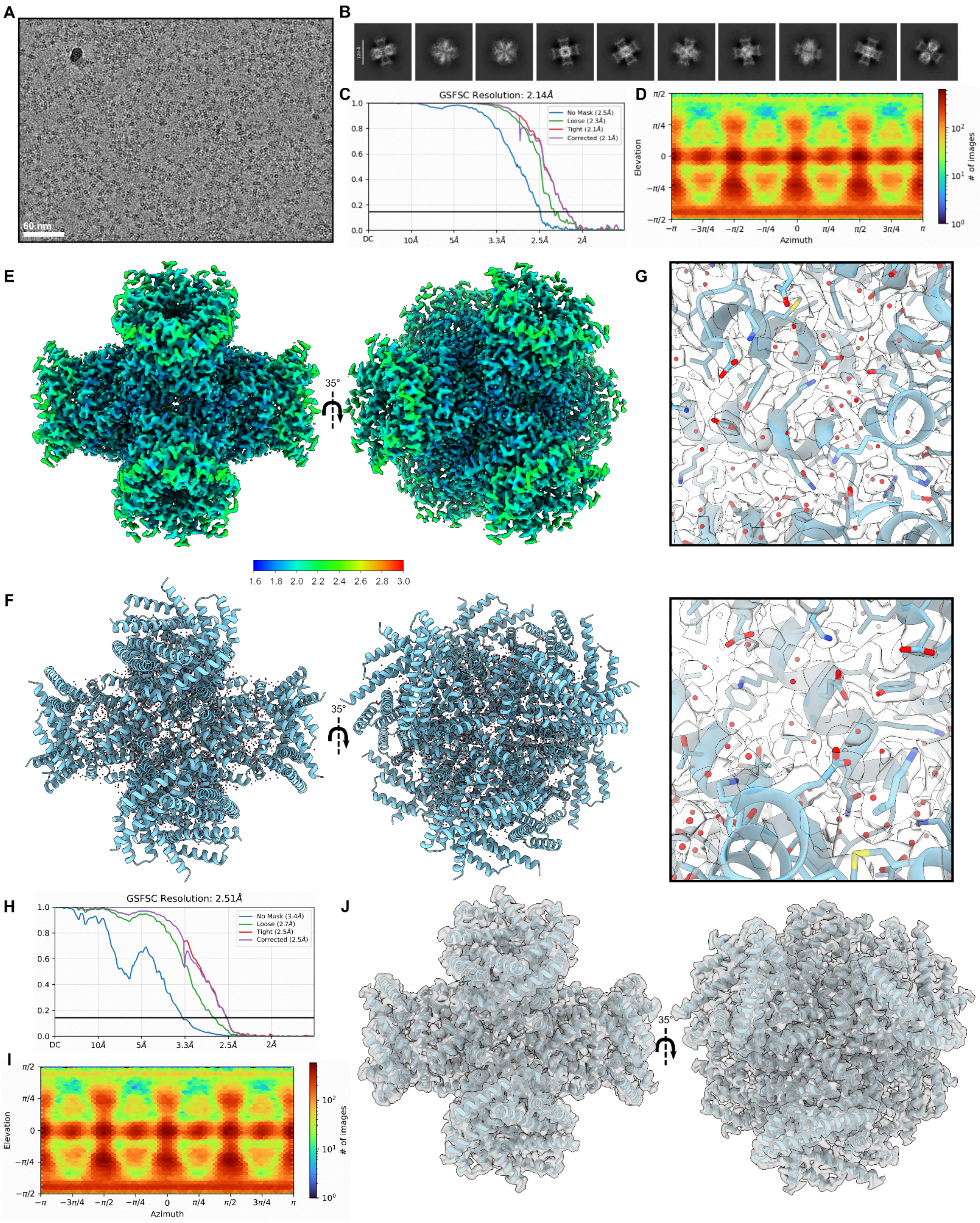

Figure S5. Cryo-EM analysis of de novo designed octahedral nanoparticle O4-102.
(**A**) Representative raw micrograph showing well-defined particles with some evidence of flocculation and aggregation. Scale bar, 60 nm. (**B**) 10 representative two-dimensional (2D) class averages displaying multiple particle orientations. (**C**) Global gold-standard Fourier shell correlation (GSFSC) curve indicating an estimated global resolution of 2.14 Å. (**D**) Orientational distribution plot demonstrating full angular sampling. (**E**) Local resolution map colored by resolution and shown along the 2- and 3- fold axes of symmetry. (**F**) Atomic model of O4-102 viewed along the 2- and 3- fold axes of symmetry, with ordered water molecules shown. (**G**) Representative map-to-model fit, shown from two angles. (**H-J**) Asymmetric refinement demonstrating a lack of symmetry-breaking features. (**H**) GSFSC curve indicating an estimated global resolution of 2.51 Å. (**I**) Orientational distribution plot demonstrating full angular sampling. (**J**) Atomic model rigid-body docked inside the unsharpened C1-refined cryo-EM density map.

bioRxiv preprint doi: https://doi.org/10.64898/2025.12.19.695620; this version posted January 2, 2026. The copyright holder for this preprint (which was not certified by peer review) is the author/funder, who has granted bioRxiv a license to display the preprint in perpetuity. It is made available under aCC-BY 4.0 International license.

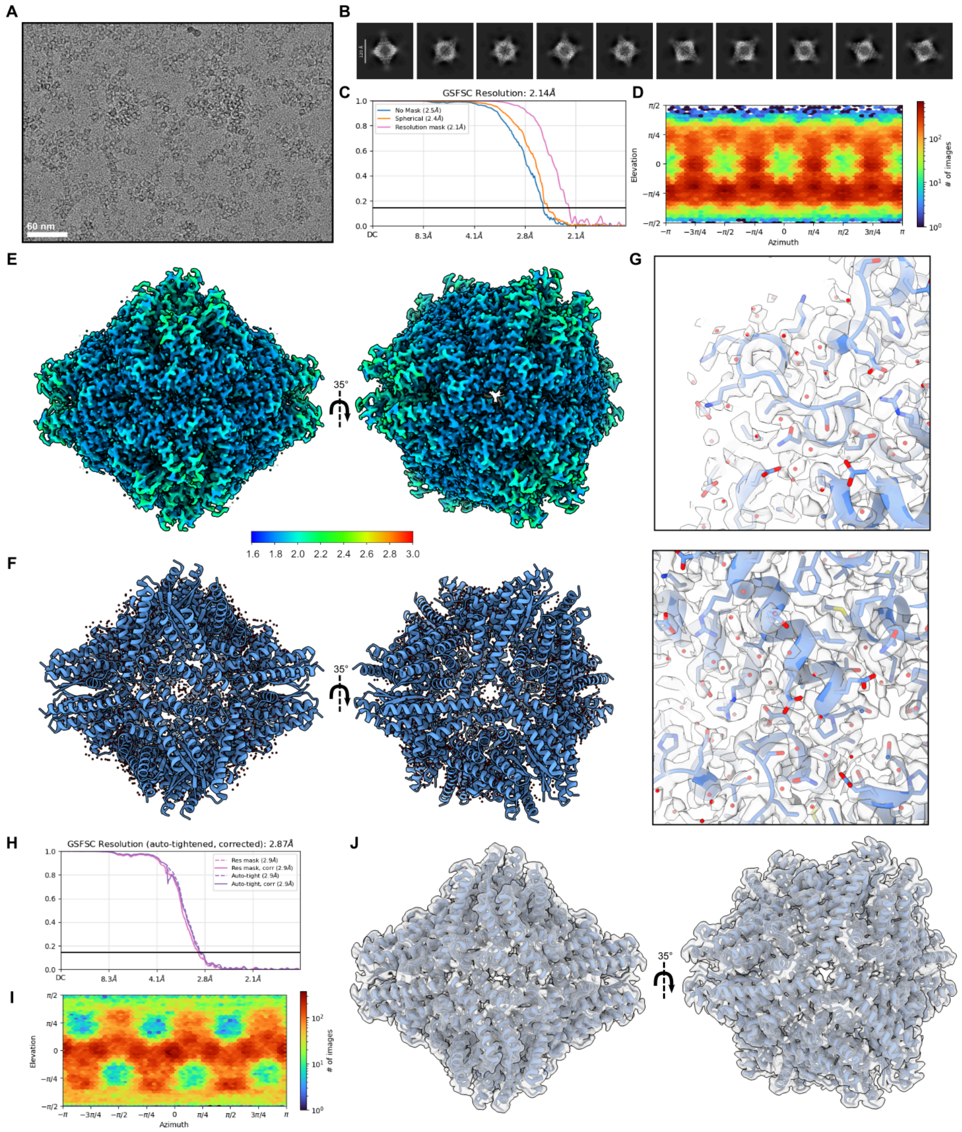

Figure S6. Cryo-EM analysis of de novo designed octahedral nanoparticle O4-104.
(**A**) Representative raw micrograph showing well-defined particles with some evidence of flocculation and aggregation. Scale bar, 60 nm. (**B**) 10 representative two-dimensional (2D) class averages displaying multiple particle orientations. (**C**) Global gold-standard Fourier shell correlation (GSFSC) curve indicating an estimated global resolution of 2.14 Å. (**D**) Orientational distribution plot demonstrating full angular sampling. (**E**) Local resolution map colored by resolution and shown along the 2- and 3- fold axes of symmetry. (**F**) Atomic model of O4-104 viewed along the 2- and 3- fold axes of symmetry, with ordered water molecules shown. (**G**) Representative map-to-model fit, shown from two angles. (**H-J)** asymmetric refinement demonstrating a lack of symmetry breaking features. (**H**) GSFSC curve indicating an estimated global resolution of 2.87 Å. (**I**) Orientational distribution plot demonstrating full angular sampling. (**J**) Atomic model rigid-body docked inside the unsharpened C1-refined cryo-EM density map.

bioRxiv preprint doi: https://doi.org/10.64898/2025.12.19.695620; this version posted January 2, 2026. The copyright holder for this preprint (which was not certified by peer review) is the author/funder, who has granted bioRxiv a license to display the preprint in perpetuity. It is made available under aCC-BY 4.0 International license.

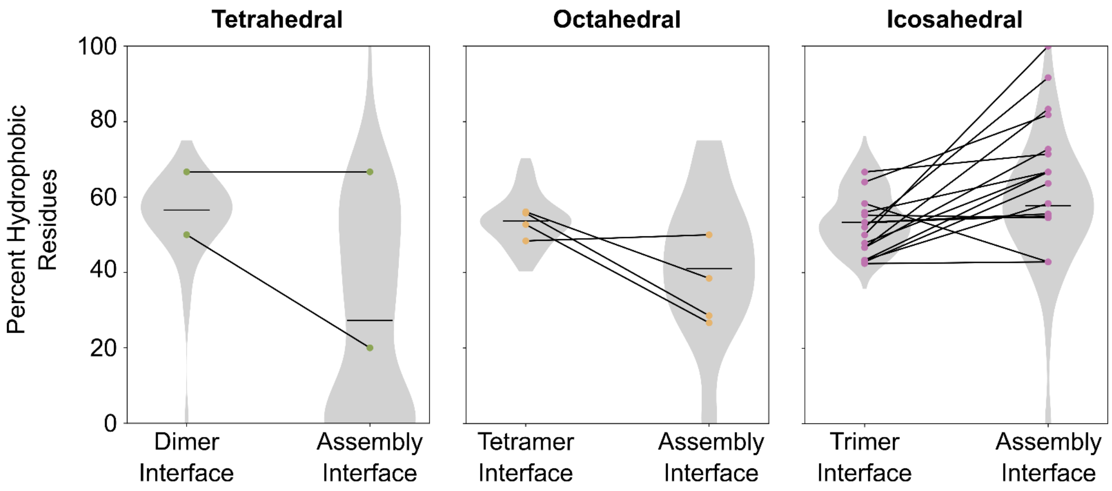

Figure S7. Analysis of hydrophobic residues at interfaces for the tetrahedral, octahedral, and icosahedral nanoparticles selected for experimental characterization.

Violin plots showing the distribution of the percent hydrophobic residues (A, V, L, I, M, F, W, P, Y) found at the oligomer and nanoparticle assembly interfaces for designs that were ordered for experimental validation (tetrahedral, n=84; octahedral, n=56; icosahedral, n=166). Assembly interfaces were defined as the set of residues including Cα atoms within 8 Å of a Cα on another chain in the design model that was not part of the same dimeric, trimeric, or tetrameric building block. Points correspond to nanoparticles that were experimentally confirmed to successfully assemble to the target architecture.

bioRxiv preprint doi: https://doi.org/10.64898/2025.12.19.695620; this version posted January 2, 2026. The copyright holder for this preprint (which was not certified by peer review) is the author/funder, who has granted bioRxiv a license to display the preprint in perpetuity. It is made available under aCC-BY 4.0 International license.

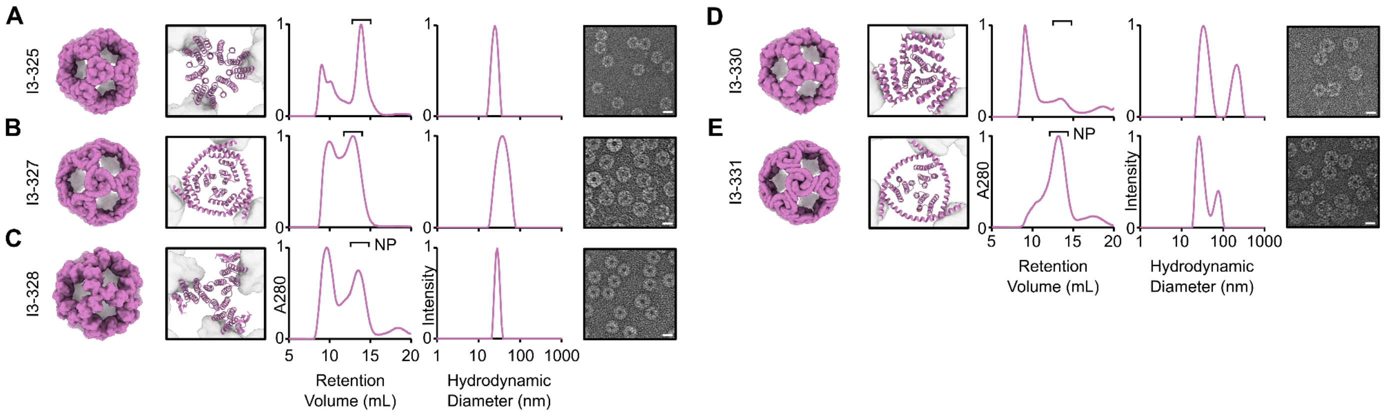

Figure S8. Characterization for additional motif-scaffolded icosahedral nanoparticles.
From left to right: Design models, SEC, DLS, and negatively stained electron micrographs for (**A**) I3-325, (**B**) I3-327, (**C**) I3-328, (**D**) I3-330, and (**E**) I3-331. The design model of the nanoparticle is shown at left and a cropped image of the design model for the diffused oligomer in the context of the nanoparticle is shown at right. nsEM scale bars = 20 nm.

bioRxiv preprint doi: https://doi.org/10.64898/2025.12.19.695620; this version posted January 2, 2026. The copyright holder for this preprint (which was not certified by peer review) is the author/funder, who has granted bioRxiv a license to display the preprint in perpetuity. It is made available under aCC-BY 4.0 International license.

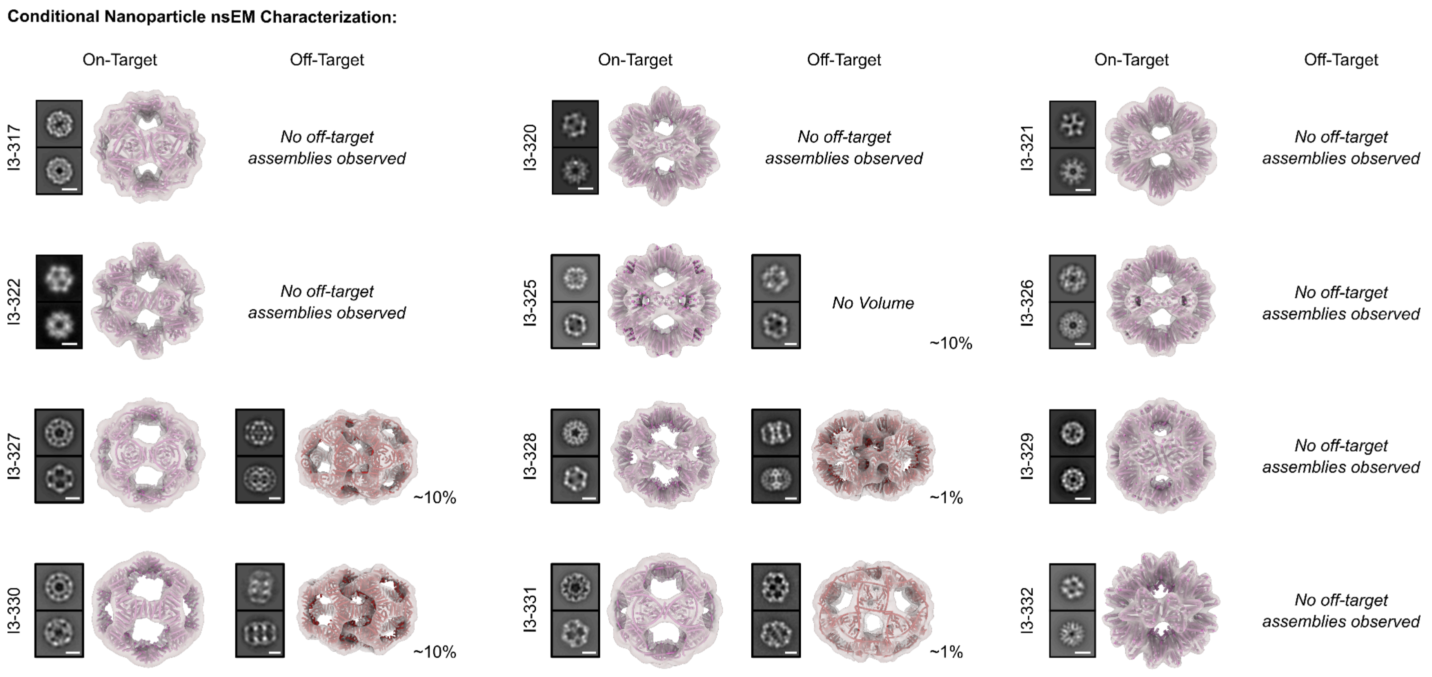

Figure S9. nsEM analysis of antigen-tailored icosahedral nanoparticles.
Left, 2D class averages and *right,* 3D reconstructions of on-target nanoparticles and off-target assemblies (when present). Reconstructions show the map in transparent gray with the model of the protein nanoparticle shown underneath as a cartoon in pink or red. Relative abundances of observed off-target species are approximated next to each species. Class averages for nanoparticles shown in Figure 4 are replicated here for clarity. Scale bars = 10 nm.

bioRxiv preprint doi: https://doi.org/10.64898/2025.12.19.695620; this version posted January 2, 2026. The copyright holder for this preprint (which was not certified by peer review) is the author/funder, who has granted bioRxiv a license to display the preprint in perpetuity. It is made available under aCC-BY 4.0 International license.

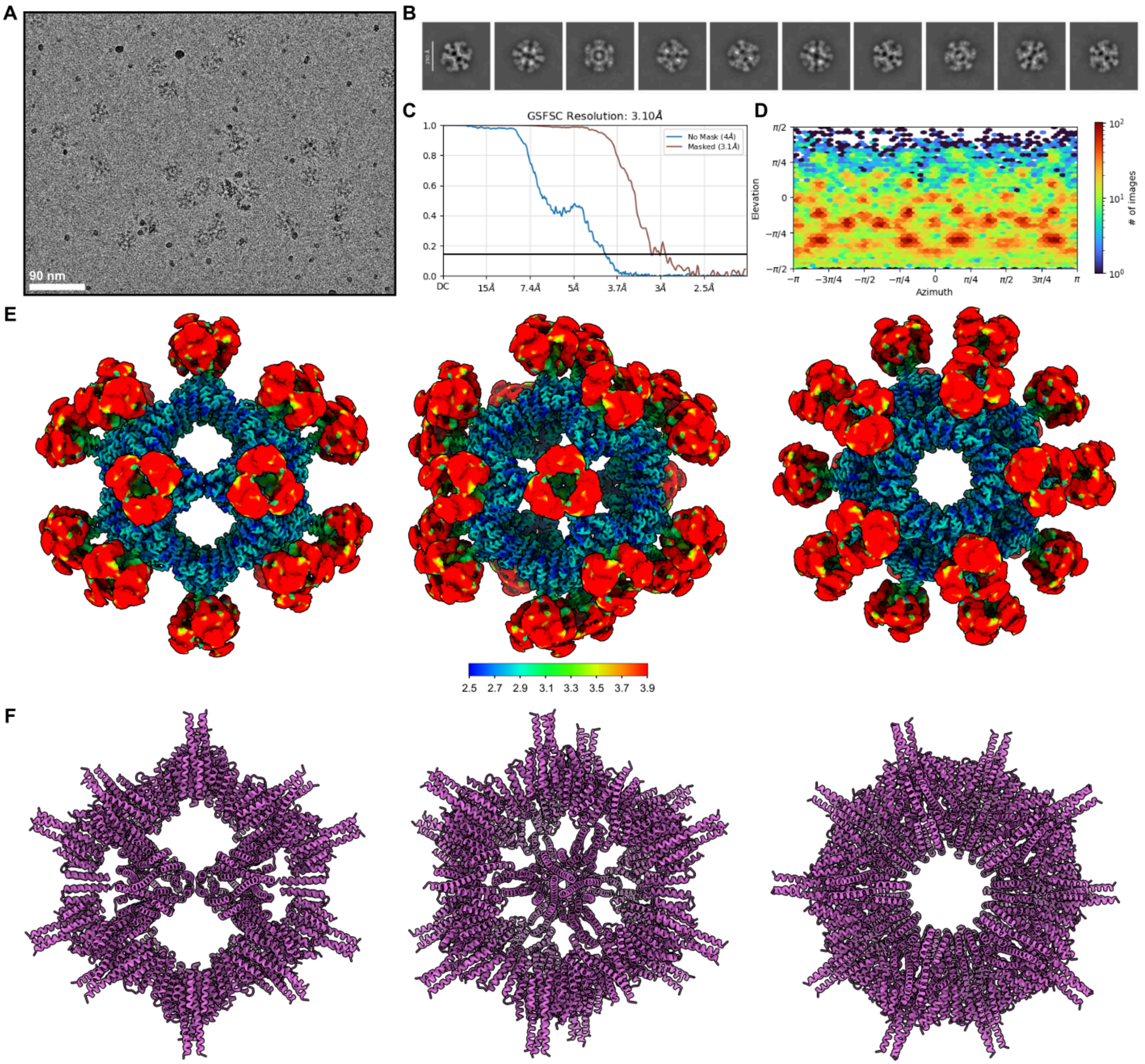

Figure S10. Cryo-EM analysis of de novo designed icosahedral nanoparticle I3-326 displaying HA trihead antigen.
(**A**) Representative raw micrograph showing well-defined particles with no evidence of flocculation or aggregation. Scale bar, 90 nm. (**B**) 10 representative two-dimensional (2D) class averages displaying multiple particle orientations. (**C**) Global gold-standard Fourier shell correlation (GSFSC) curve indicating an estimated global resolution of 3.10 Å. (**D**) Orientational distribution plot demonstrating near-full angular sampling. (**E**) Local resolution map colored by resolution and shown along the three principal symmetry axes. (**F**) Cryo-EM density and atomic model of I3-326 viewed along the three major symmetry axes.

bioRxiv preprint doi: https://doi.org/10.64898/2025.12.19.695620; this version posted January 2, 2026. The copyright holder for this preprint (which was not certified by peer review) is the author/funder, who has granted bioRxiv a license to display the preprint in perpetuity. It is made available under aCC-BY 4.0 International license.

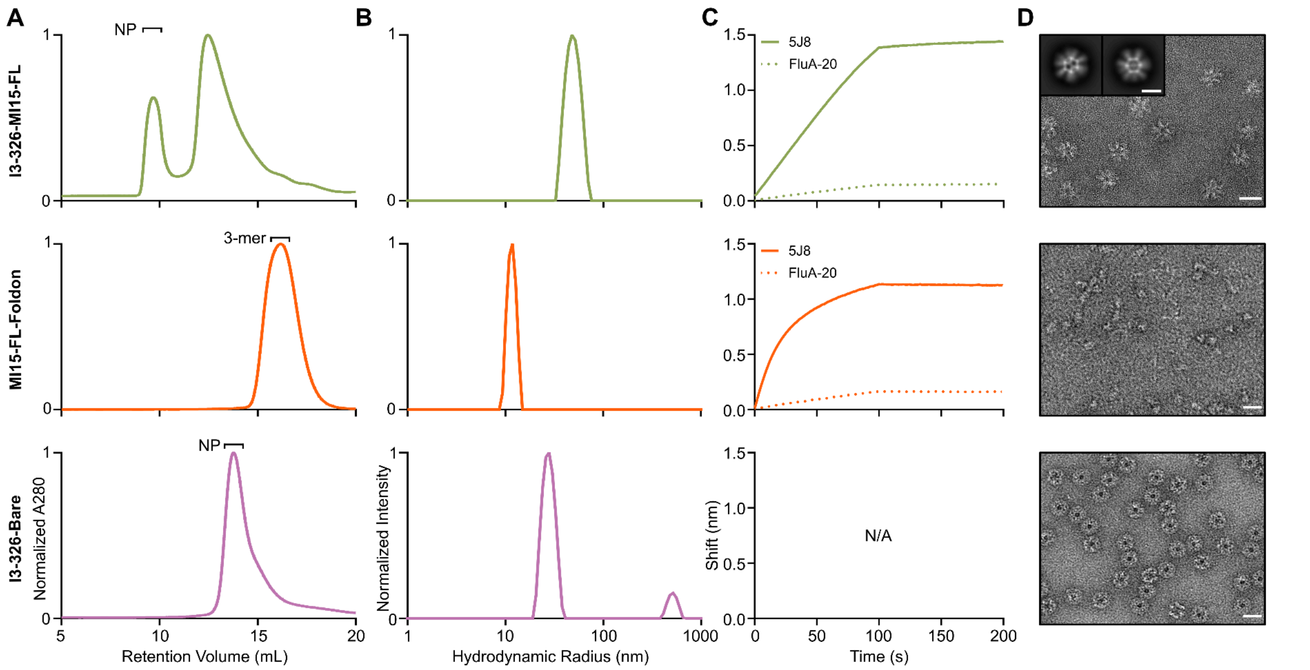

Figure S11. Characterization of proteins used in immunogenicity study.
(**A**) SEC, (**B**) DLS, (**C**) BLI, and (**D**) nsEM for the proteins used in the immunogenicity study. SEC and DLS plots are normalized and fractions purified for further characterization are indicated (“NP”, nanoparticle; “3-mer”, trimer). 2D class averages are shown for I3-326-MI15-FL. Scale bars = 50 nm, 20 nm, and 20 nm for I3-326-MI15-FL, MI15-FL-Foldon, and I3-326-Bare, respectively. Scale bar = 25 nm for I3-326-MI15-FL 2D class averages.

bioRxiv preprint doi: https://doi.org/10.64898/2025.12.19.695620; this version posted January 2, 2026. The copyright holder for this preprint (which was not certified by peer review) is the author/funder, who has granted bioRxiv a license to display the preprint in perpetuity. It is made available under aCC-BY 4.0 International license.

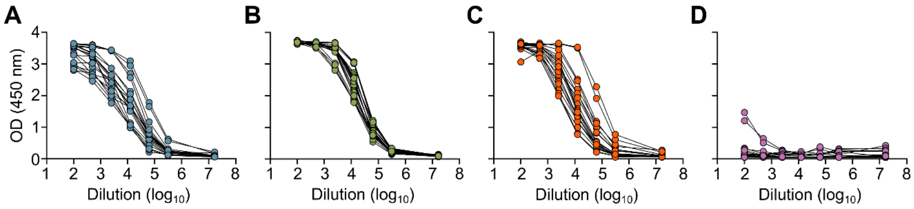

Figure S12. Raw ELISA data and fits used to determine titers shown in Figure 5.
The ELISA curves showing week six serum IgG binding to full-length HA ectodomain trimer (A/Michigan/45/2015) for (**A**) I3-326-MI15-TH, (**B**) I3-326-MI15-FL, (**C**) MI15-FL-Foldon, and (**D**) I3-326-Bare.

bioRxiv preprint doi: https://doi.org/10.64898/2025.12.19.695620; this version posted January 2, 2026. The copyright holder for this preprint (which was not certified by peer review) is the author/funder, who has granted bioRxiv a license to display the preprint in perpetuity. It is made available under aCC-BY 4.0 International license.

Table S1. Amino Acid Sequences

Construct	Sequence*
I3-297	MSGSEEQERRQRIVRMIAEELTEEFLAEYKLFNYEEVKSVKTEFLQYRDVSIVLVTIETVKATIKVIIVVAGGSTSVEELETLIRLIMKLTNSPIIATIIDEDPVLARLKALRLKEIAKEEGKTILVKLRSLEEAEEELEIREKAEEILEKEK**GSWELQGSHHHHHH**
I3-298	MSGKEELKEIVEFVEETVTEVLKFIKEVVKLLEMREKSRLPEVKEAIADAVELTIKLNNDETVKLMVELIFSLLGLNEEEREKYTKLVFESVKEKIAEIAARREAGDKSLKMEDVANEVLSTIFSTLYKKLKEEIEKKEEEIKKKAEEIKKKI**GSWELQGSHHHHHH**
I3-299	MSGAKEKLVVSISLSGSLTEEDKEKVLRAIEDILDMLSEMKDAEKYLVVVFSRVDVEFAEEVLEIAVEAKKKGILKDIHVVTTSVSNYAVAKKYSKKEGVKVSLATSGLMALNTAVVEIAKLKPDKIAGFSFRKGTVEETITKSYLILLEIKE**GSWELQGSHHHHHH**
I3-301	MSGMKKVTLTFVKGAKEETILELVKRAAEAGVDEITIELIDVALTYETIKKILEIAKSAKKVNITIDLGTDPVASSATLLNLLLALGAKEAAKIVASYLAYVAAGNEEKANEKLLELAEKLVTELGADITITVKEENGKTVVKLKVTITIENK**GSWELQGSHHHHHH**
I3-302	MSGMKTVTVHLTDTRALVRALLAGADRIVVVSVIRTLEELEENLELLKAVLKIAKAAGVEVDVVVLLIGEEVKERAEEIRAEIEAWLAEVGEKGEVYIEEVPTLAEARLLEGLAELGLDVLVAAIKIIKNVKKTEERLENLTIILITALKLKK**GSWELQGSHHHHHH**
I3-304	MSGSEEEERRREIVRLVAEERTERFLAEYKLVNYGLVKSVTTGFAQVGDVAVVSVTITTTVETIKVIHLVAGGATSVEELVGLVRALMNAAQAPLVVTIIDEDEVRAELKALRIREVAKELGQTVLVELLRLEEAERRAEVRRRARERLEKER**GSWELQGSHHHHHH**
I3-306	MSGAERNLTLTLRSDLSNAVEALALVVKLVPRVDTITVVGISGSSVEEFERITATLEAAVELLAPVAKEAGVKELTVELILENMEITPEILARIEALMRRLAAIPGVNVTVSILNDGVNITIGQDLSVEEAIALLREAVAKGIGITVTVRQRE**GSWELQGSHHHHHH**
I3-307	MSGMKTNLTLTVSNDVQALAAKIIIEKTKFDEITITLRNVSPTSVKLLKEALVKNKSKITIHLTDNSEELITEELKKELTEMVKALAKKGNEIELVVTASEVELVKIATEMVKALAKEGKDIKVTIHFDASDEKVLEVMEAILELAKTIEELN**GSWELQGSHHHHHH**
I3-308	MSGMEEFKEELRIVAEVVLEELEERAALALANSLDTAEVRTVMVEIAIKRTIVRELSRLIPELEELREIAERLDAILDEMLDAVFARDPAEAAVLMRAILEARDKVEELLSLISRRYKGEERLRILTELLLKDPLEIRRELRKFRIEERKEER**GSWELQGSHHHHHH**
I3-310	MSGEELKEKVEVLITKLLTHWMKTLLEEFTKAVEAVKFLKETLKDDDEALLKALKILLEAMLKRIEKKLKEFAKTIKVPEEYKPIAEKIEETLIKITKEVLEEEKDFIITVLLFAAERPDVSTNVIVTLVMTLGREIHKKLKEEVEKLLKELE**GSWELQGSHHHHHH**
I3-311	MSGEELRLKVEVALTKLMTHWMETLHKEFTKALKAVKALMEALANDDEALLKALRVLLEGMLKNIEAAIKEFAKTLKWPEEYQPILDKITEALVEITREVLEERRLYILTVLLYAAKNPEVAENVITTLVMTLGEEIFERLEEWVEKYLKELE**GSWELQGSHHHHHH**
I3-312	MSGMKEITLNFSVTNNPAVLAAITYAVKDLKVPINVSLTFTEDDEEFVKVFEELLKSVKNIKKVTVTLYGGVSPESAKRIMEALAKLKGVEITIVLENVSILTTLVIAEELKKIMDVSNLTVEIVNNKAENAAQTAATIFIITNMIKEYEEER**GSWELQGSHHHHHH**

bioRxiv preprint doi: https://doi.org/10.64898/2025.12.19.695620; this version posted January 2, 2026. The copyright holder for this preprint (which was not certified by peer review) is the author/funder, who has granted bioRxiv a license to display the preprint in perpetuity. It is made available under aCC-BY 4.0 International license.

I3-313	MSGIPLLLLLKIKKLYEEMKKNEEEIELYLIALNLVPEEYKPEVKLKIVEALFKYYLLKISLVPEEKKPIAMEVVAAIIRKAGDPSMTPSKYEALKELFELMLELIDVGLPREKVLKLLEESLKELEESGHKSKFVTYAKELAIKTLEEYKKE**GSWELQGSHHHHHH**
I3-314	**MSGHHHHHHGSWELQGS**SEENQLLLRFGVKAVAYKQFKDVEELKPLVEEYAKWIKENAVKIMKIILKKLIEILKDKSITNEEKALKAQELVTIAKLVVDAMKELGEDVSELVSILNNMLLLLATEADIVKSSLLAMFEILLDTLKHAGKTAELGRFVISQIEILLDV
I3-315	**MSGHHHHHHGSWELQGS**SREIMELILEIAEKLLKLEKLGMSPLLVNTLEQLALLSPDEKLPDVLKLLELLVKALEEKDEEKKLELLKKAFEIIVKKFLPLHPRASTFLLLRLTYEYFELATKAALELMEELDRELAEILARILLSVAKIKNKELAPLFQALLAAIER
I3-316	**MSGHHHHHHGSWELQGS**MKRVVVHNDDIGRLIRALLEGADEIVVISVIRTLEELEENLTLIEAIIEIAKDAGVEVHIIIIAIGEEVKEKLEEIKKKVEEILKKYGQKGKAYFIEVETLAEAKLLEGLAEIGLDVAIAAMEIIKNEKDVEKRLENLLIILKTALKLKK
I3-317	**MSGHHHHHHGSWELQGS**GSSSLVYYLIKIFLKIAEALPELKEEIKTLIIKMAVDAGYEAEAFRVLAEAGDEEGLKLLVRELKRKLVREALETGDLEELKKVPERLLERLREIAERVREEARRALELAKRALELLEEVEDEELRELLRELLEELLREAAELLRVEERIERLLKEEEK
I3-320	**MSGHHHHHHGSWELQGS**GSGELTKALAESNLIELVAVAAELMEERREEFVRRVIEKMSEILPEELVKATLEVLATRGLLSDELMLRVAELYPEIAPHIIRELTRREYLSKEERELLARLRLLRRQLYVYLDIAVELGAISPEEAEKIKAFVEELEEEVERYIEEKVKRRKEERRKE
I3-321	**MSGHHHHHHGSWELQGS**GSKELALNLAYTNLALIPVTDIVTSEILKINAMNYMDLAGLSEEEKKYFKELADKLIELSKKKRKLEIEIYKILMEVANKEKMKKLHLEALLEDIEIEALKLFVLTLVDPERVEEVLKEAKEEIAEKAPEEVKELALKYFEELSKRKIEEAKKLKEKRE
I3-322	**MSGHHHHHHGSWELQGS**GSRALARELVGTVASLAAVTGRRSEVLTALFLIDEVEEHREELPEEEVELLEKLREALKKKLELLLKIAPERLKKLEEEAEKLFKEFLELLLKSETEEDLIKMREKLIELVNILIDLLVEALDALRLGDPLVEEYKKAAEKIRERVEAALKLLEYKEKE
I3-325	**MSGHHHHHHGSWELQGS**GSPLREFREALDAIAKQVVREGISPELVERFLATMVALAKAGDLEGLNTMRELAKVGLLEIGILIAEGASDEEVLRALIADMTRRLVRYAERHPERAGEAVRAAGELLNLVLENKDKLPISDVIIIAGRALDAVLEEGMSPETAIELAKLELAAVLALH
I3-326	**MSGHHHHHHGSWELQGS**GSPLREFREALDAIAKQVVREGISPELVKRFLETMVKLAKAGDLEGLNTMRELAKVGLLEIGILIAEGASDEEVLRALIADMTRRLVRYAERHPERAGEAVRAAGELLNLVLENKDKLPISDVIIIAGRALDAVLEEGMSPETAIELAKLELEAVLELH
I3-327	**MSGHHHHHHGSWELQGS**GSAELALNLYYFAEELKLPKEFVAKFLIEALKKIEELKKKDQLELLKKALKIIKEEKDLDLLLKAKLTALVLKQLPELKLLAELLETLVELYELEKDPEERELLEELIRYVLTRFKTKLPLVEKYLKEEEEKLEKEEKEELEKLKKKLKELLKKLIEEE
I3-328	**MSGHHHHHHGSWELQGS**GSMKLALAIARATLRKLAGEDREAVDIELARTAALIAIEEGAPLEEVLELLRLLVRAMDALAERGAPLELVLAVFDALIDIILASKELEMTVEVTALVAMAADTLARRAAELGRAVTITIRETPENKEKVEAIVRILETVAALYASEPGLRITIEVVKL
I3-329	**MSGHHHHHHGSWELQGS**GSRLLAEKLVLTLTEVLIELAKLSKDKELKDRLILLAFDIVKLAAEVGKEVAVRVAYTLMVELKLSLAEQKYLLVLLAREVGLSAEELRLIADAVAEANNLDELTKKRILATIEALERDPEATARIVEAEFELEHAVALGRRRLRDARADALLAELEAL
I3-330	**MSGHHHHHHGSWELQGS**GSDKLVEELEALLVLKLVRKLRRAAELVGEENLATIFGILDDLLKEAILELDEEKIEEAKKKLVAMFKTLEKKYPELAKLNELFLLYDGLDMLEEREEVEELMARYRKLLASMPASELISLFSSLATFIMDLLGKPETEKLLLIGKLTVAIAKLKLEEA

bioRxiv preprint doi: https://doi.org/10.64898/2025.12.19.695620; this version posted January 2, 2026. The copyright holder for this preprint (which was not certified by peer review) is the author/funder, who has granted bioRxiv a license to display the preprint in perpetuity. It is made available under aCC-BY 4.0 International license.

I3-331	**MSGHHHHHHGSWELQGS**GSAELARLLAIELRDVLSEESVLRFARLSIREAIREYVARGDYAEALRFVLDVVEELKELGISEEGILEMLWEVAREFIREGDERGLRIIVGAMLKVGVPVEELIARLARLRLEVEKEAAEEAKRREEWEKEYEELEKVVEGVLKGTKALVAEVKAILA
I3-332	**MSGHHHHHHGSWELQGS**GSTRLEIALIRNEVLKALLADEREAIRYLYFALRLVTTLEGAKTLAELLKLAADLDENFKIALELVELRIKIEEMSPEERLENEELYDKAIELLKELSDRLKETIILETGERIAKLREEGNPEAELEAKRTLVAIKVIDLETEEEIEEIEKILKEDKEK
O4-101	MSGMKGEIGGELFKEAEKNKLLLETLEKDIKAKRALTVGLLLDFTETASRTFRFLGRAAGLPEELIEEALERLEEITAEHLRKIMDPEYAKKHTTEEVIKEFEEDVLEWAEEREEIRDIVETVILLARYGTPALTLYKELEFNKLLLELMAKEGS**GSWELQGSHHHHHH**
O4-102	MSGGEATELLIMTRIETDIKYMLLEGKPVEEIEKFFTDTTSATSILGGHIQKHLELSKLEAEKELPETYPKALEKLKPLIERKVKVKALELEEVMKLMGKPEEEIEEYRKLFEELLKKAKTPIELLALLEYLEEELKKAKLELAKKWVEEAKKGS**GSWELQGSHHHHHH**
O4-103	MSGMEKFEEAREFLKRYHRGVYIAKMRRARKEFETRLEKAEDPNSEEMKRERRLLEDRLAVRRLVEEGDLVSAARYYAALLRLGAPEEHLEAVEETLLEEIRERLEELEDENMLKLLMGTLGVPEELAKEISIELRLELRTALAEAMVELDRLILELREEGAPEEHIRRLEELRERLGEILKEGS**GSWELQGSHHHHHH**
O4-104	MSGPINIVRVVPELLSLIAVAIAKEAEKKLKEAGKDVKIEVLEAPLSGGVSQYRLVEFAKKKGLSPEQLGQMMGTIRRVLGARLEEIQRRQALFLLEHSLEEAKELSLEYIRLLLAAEGVVTPETAELIVEFFRWYIEELEKEFEELVKKIVEGS**GSWELQGSHHHHHH**
T2-10	MNKEVIKKIAKALEEIAKALEKLSDEDKLLLNALNQVAMAYQTEALAAIAKMIAEGESIEEIRKKIEEIAEKMKKALKKMIELLKTDEEAAKLALKAQTKKMLKATQELLEYIEILLHAVFTDDEILDLNESNILALKALAEALKLWAEL**GSGSGSGSLEHHHHHH**
T2-71	MMSPREKRRELIKKFVEKDRETARLLTRRMRREVLRLELLELLDEEAKRSKEDETLKLTLLLMKIYVELGDWEEAEELSELLPDEIQLALRETREELEELERRWLELSDEELVELLLENYERAEELEKRGYVTPELRLLLAALRAMLEEAA**GSGSGSGSLEHHHHHH**
I3-326-MI15-FL	(MDSKGSSQKGSRLLLLLVVSNLLLPQGVLA)DTLCIGYHANNSTDTVDTVLEKNVTVTHSVNLLEDKHNGKLCKLRGVAPLHLGKCNIAGWILGNPECESLSTASSWSYIVETSNSDNGTCFPGDFINYEELREQLSSVSSFERFEIFPKTSSWPNHDSNKGVTAACPHAGAKSFYKNLIWLVKKGNSYPKLNQSYINDKGKEVLVLWGIHHPSTTADQQSLYQNADAYVFVGTSRYSKKFKPEIATRPKVRDQEGRMNYYWTLVEPGDKITFEATGNLVVPRYAFTMERNAGSGIIISDTPVHDCNTTCQTPEGAINTSLPFQNIHPITIGKCPKYVKSTKLRLATGLRNVPSIQSRGLFGAIAGFIEGGWTGMVDGWYGYHHQNEQGSGYAADLKSTQNAIDKITNKVNSVIEKMNTQFTAVGKEFNHLEKRIENLNKKVDDGFLDIWTYNAELLVLLENERTLDYHDSNVKNLYEKVRNQLKNNAKEIGNGCFEFYHKCDNTCMESVKNGTYDYPKYSEEAKLNREKIDGVGSELAYLLGLDEFREALDAIAKQVVREGISPELVKRFLETMVKLAKAGDLEGLNTMRELAKVGLLEIGILIAEGASDEEVLRALIADMTRRLVRYAERHPERAGEAVRAAGELLNLVLENKDKLPISDVIIIAGRALDAVLEEGMSPETAIELAKLELEAVLELH**GGWELQHHHHHH**
I3-326-MI15-TH	(MDSKGSSQKGSRLLLLLVVSNLLLPQGVLA)VAPLHLGKCNIAGWILGNPECESLSTASSWSYIVETSNSDNGTCFPGNFINYEELRCQLSSVSSFERFEIFPKTSSWPNHDSNKGVTAACPHAGAKSFYKNLIWLVKKGNSYPKLNQSYINDKGKEVLVLWGIHHPSTTADQQSLYQNEDTYVFVSTSRYDKVFKPIIATRPKVRDQEGRMNYYWTLVEPGDKITFEATGNLVVPRYAFTMERNAGSGSGSCIENINSKIYHIENEIAELAYLLGLDEFREALDAIAKQVVREGISPELVKRFLETMVKLAKAGDLEGLNTMRELAKVGLLEIGILIAEGASDEEVLRALIADMTRRLVRYAERHPERAGEAVRAAGELLNLVLENKDKLPISDVIIIAGRALDAVLEEGMSPETAIELAKLELEAVLELH**GSHHHHHH**

bioRxiv preprint doi: https://doi.org/10.64898/2025.12.19.695620; this version posted January 2, 2026. The copyright holder for this preprint (which was not certified by peer review) is the author/funder, who has granted bioRxiv a license to display the preprint in perpetuity. It is made available under aCC-BY 4.0 International license.

Full-length HA ectodomain trimer (A/Michigan/45/2015) on T4 foldon trimerization domain.	(MDSKGSSQKGSRLLLLLVVSNLLLPQGVLA)DTLCIGYHANNSTDTVDTVLEKNVTVTHSVNLLEDKHNGKLCKLRGVAPLHLGKCNIAGWILGNPECESLSTASSWSYIVETSNSDNGTCFPGDFINYEELREQLSSVSSFERFEIFPKTSSWPNHDSNKGVTAACPHAGAKSFYKNLIWLVKKGNSYPKLNQSYINDKGKEVLVLWGIHHPSTTADQQSLYQNADAYVFVGTSRYSKKFKPEIATRPKVRDQEGRMNYYWTLVEPGDKITFEATGNLVVPRYAFTMERNAGSGIIISDTPVHDCNTTCQTPEGAINTSLPFQNIHPITIGKCPKYVKSTKLRLATGLRNVPSIQSRGLFGAIAGFIEGGWTGMVDGWYGYHHQNEQGSGYAADLKSTQNAIDKITNKVNSVIEKMNTQFTAVGKEFNHLEKRIENLNKKVDDGFLDIWTYNAELLVLLENERTLDYHDSNVKNLYEKVRNQLKNNAKEIGNGCFEFYHKCDNTCMESVKNGTYDYPKYSEEAKLNREKIDGVGSGYIPEAPRDGQAYVRKDGEWVLLSTFL**<u>GSGLNDIFEAQKIEWHEGHHHHHH</u>**
Full-length HA ectodomain trimer (A/Michigan/45/2015) on I53_dn5B nanoparticle component (*55*).	(MKAILVVLLYTFTTANA)DTLCIGYHANNSTDTVDTVLEKNVTVTHSVNLLEDKHNGKLCKLRGVAPLHLGKCNIAGWILGNPECESLSTASSWSYIVETSNSDNGTCFPGDFINYEELREQLSSVSSFERFEIFPKTSSWPNHDSNKGVTAACPHAGAKSFYKNLIWLVKKGNSYPKLNQSYINDKGKEVLVLWGIHHPSTTADQQSLYQNADAYVFVGTSRYSKKFKPEIATRPKVRDQEGRMNYYWTLVEPGDKITFEATGNLVVPRYAFTMERNAGSGIIISDTPVHDCNTTCQTPEGAINTSLPFQNIHPITIGKCPKYVKSTKLRLATGLRNVPSIQSRGLFGAIAGFIEGGWTGMVDGWYGYHHQNEQGSGYAADLKSTQNAIDKITNKVNSVIEKMNTQFTAVGKEFNHLEKRIENLNKKVDDGFLDIWTYNAELLVLLENERTLDYHDSNVKNLYEKVRNQLKNNAKEIGNGCFEFYHKCDNTCMESVKNGTYDYPKYSEEAKLNREKIDGVSAEEAELAYLLGELAYKLGEYRIAIRAYRIALKRDPNNAEAWYNLGNAYYKQGRYREAIEYYQKALELDPNNAEAWYNLGNAYYERGEYEEAIEYYRKALRLDPNNADAMQNLLNAKMREE**<u>GGWELQHHHHHH</u>**
HA-ferritin nanoparticle (A/Michigan/45/2015)	(MDSKGSSQKGSRLLLLLVVSNLLLPQGVLA)DTLCIGYHANNSTDTVDTVLEKNVTVTHSVNLLEDKHNGKLCKLRGVAPLHLGKCNIAGWILGNPECESLSTASSWSYIVETSNSDNGTCYPGDFINYEELREQLSSVSSFERFEIFPKTSSWPNHDSNKGVTAACPHAGAKSFYKNLIWLVKKGNSYPKLNQSYINDKGKEVLVLWGIHHPSTTADQQSLYQNADAYVFVGTSRYSKKFKPEIATRPKVRDQEGRMNYYWTLVEPGDKITFEATGNLVVPRYAFTMERNAGSGIIISDTPVHDCNTTCQTPEGAINTSLPFQNIHPITIGKCPKYVKSTKLRLATGLRNVPSIQSRGLFGAIAGFIEGGWTGMVDGWYGYHHQNEQGSGYAADLKSTQNAIDKITNKVNSVIEKMNTQFTAVGKEFNHLEKRIENLNKKVDDGFLDIWTYNAELLVLLENERTLDYHDSNVKNLYEKVRNQLKNNAKEIGNGCFEFYHKCDNTCMESVKNGTYDYPKYSEEAKLNREKIDSGGDIIKLLNEQVNKEMQSSNLYMSMSSWCYTHSLDGAGLFLFDHAAEEYEHAKKLIIFLNENNVPVQLTSISAPEHKFEGLTQIFQKAYEHEQHISESINNIVDHAIKSKDHATFNFLQWYVAEQHEEEVLFKDILDKIELIGNENHGLYLADQYVKGIAKSRKS

*Signal peptides are indicated in parentheses and purification/solubility/cleavage tags are bolded and underlined.

bioRxiv preprint doi: https://doi.org/10.64898/2025.12.19.695620; this version posted January 2, 2026. The copyright holder for this preprint (which was not certified by peer review) is the author/funder, who has granted bioRxiv a license to display the preprint in perpetuity. It is made available under aCC-BY 4.0 International license.

Table S2. CryoEM Data Collection, Processing, and Modeling Statistics (On-Target)

	I3-304<br>PDB: 9ZQI<br>EMDB: 74566	O4-102<br>PDB: 9ZOJ<br>EMDB: 74498	O4-104<br>PDB: 9ZPM<br>EMDB: 74530	I3-326<br>PDB: 9ZSD<br>EMDB: 74706
**Data Collection**				
Microscope	Titan Krios	Titan Krios	Titan Krios	Titan Krios
Voltage (kV)	300	300	300	300
Detector	Gatan K3	Gatan K3	Gatan K3	Gatan K3
Energy Filter	Gatan BioQuantum Gif	Gatan BioQuantum Gif	Gatan BioQuantum Gif	Gatan BioQuantum Gif
Recording mode	Counting	Counting	Counting	SuperResolution
Magnification	105,000 X	105,000 X	105,000 X	81,000 X
Movie micrograph pixel size (Å)	0.83	0.83	0.83	0.5305
Dose rate (e^-/Å^2/s)	10.6	9.26	9.20	16.15
Frames per movie micrograph	99	99	99	75
Frame exposure time (s)	0.0505	0.0505	0.0505	0.0413
Movie micrograph exposure time (s)	5.009	5.009	5.009	3.096
Total dose (e^-/Å^2)	52.9	46.29	46.08	50
Under focus range (μm)	0.8 - 1.8	0.8 - 1.8	0.8 - 1.8	0.8 - 1.8
No. of movie micrographs	6,302	6,375	11,590	4,091
**Map Processing**				
Symmetry Applied	I	O	O	I
Extraction Box Size (pix)	500	600	500	1400
Initial particle images (no.)	1,321,177	728,534	2,201,438	935,578
Final particle images (no.)	350,549	320,100	402,797	38,017
Map resolution (Å)	2.40	2.14	2.14	3.10
FCS threshold	0.143	0.143	0.143	0.143
Map resolution range (Å)	2.3 - 3.7	1.8 - 2.7	1.8 - 2.3	2.5-2.9
**Refinement**				
Initial model used	Design Model	Design Model	Design Model	Design Model
Map resolution (Å)	2.40	2.14	2.14	3.10
FCS threshold	0.143	0.143	0.143	0.143
Model resolution range (Å)	2.35 - 2.50	1.8 - 2.4	1.8 - 2.3	2.5-2.9
Map sharpening B factor	-115.1	-76.9	-83.0	-127
Model composition				
Non-hydrogen atoms	66,300	28,968	30,870	85,680
Protein Residues	9,000	3,600	3,600	11,100
Ligands	Na	Na	Na	Na
Water	2,161	2,556	2,022	Na
B factors (Å)				
Protein	49.27	26.48	32.02	88.40
Ligands	Na	Na	Na	Na
R.M.S. deviations				
Bond lengths (Å)	0.002	0.011	0.010	0.003
Bond angles (°)	0.445	1.798	1.265	0.561
Validation				
MolProbity score	1.00	1.29	0.81	1.37
Clashscore	2.25	5.37	0.66	2
Rotamer Outliers (%)	0.75	0.00	1.32	3.43
Ramachandran plot				
Favored (%)	99.07	99.44	99.27	99.33
Allowed (%)	0.60	0.56	0.73	0.13
Disallowed (%)	0.34	0.00	0.00	0.55

bioRxiv preprint doi: https://doi.org/10.64898/2025.12.19.695620; this version posted January 2, 2026. The copyright holder for this preprint (which was not certified by peer review) is the author/funder, who has granted bioRxiv a license to display the preprint in perpetuity. It is made available under aCC-BY 4.0 International license.

Table S3. CryoEM Data Collection, Processing, and Modeling Statistics (Additional I3-304 State)

	Off-Target-I3-304<br>PDB: 9ZOL<br>EMDB: 74499	
**Data Collection**		
Microscope	Titan Krios	
Voltage (kV)	300	
Detector	Gatan K3	
Energy Filter	Gatan BioQuantum Gif	
Recording mode	Counting	
Magnification	105,000 X	
Movie micrograph pixel size (Å)	0.83	
Dose rate (e^{-}/Å^{2}/s)	10.6	
Frames per movie micrograph	99	
Frame exposure time (s)	0.0505	
Movie micrograph exposure time (s)	5.009	
Total dose (e^{-}/Å^{2})	52.9	
Under focus range (μm)	0.8 - 1.8	
No. of movie micrographs	6,302	
**Map Processing**		
Symmetry Applied	D5	C1
Extraction Box Size (pix)	500	
Initial particle images (no.)	8,592	
Final particle images (no.)	6,952	
Map resolution (Å)	3.99	7.33
FCS threshold	0.143	
Map resolution range (Å)	6.70 - 4.32	8.66 - 5.02
**Refinement**		
Initial model used	Design Model	
Map resolution (Å)	3.99	
FCS threshold	0.143	
Model resolution range (Å)	4.85 - 3.75	
Map sharpening B factor	78.2	503.0
Model composition		
Non-hydrogen atoms	33,720	
Protein Residues	5,890	
Ligands	Na	
B factors (Å)		
Protein	142.30	
Ligands	Na	
R.M.S. deviations		
Bond lengths (Å)	0.003	
Bond angles (°)	0.481	
Validation		
MolProbity score	1.20	
Clashscore	4.17	
Rotamer Outliers (%)	0.83	
Ramachandran plot		
Favored (%)	98.62	
Allowed (%)	1.38	
Disallowed (%)	0.00	

bioRxiv preprint doi: https://doi.org/10.64898/2025.12.19.695620; this version posted January 2, 2026. The copyright holder for this preprint (which was not certified by peer review) is the author/funder, who has granted bioRxiv a license to display the preprint in perpetuity. It is made available under aCC-BY 4.0 International license.

Table S4. Crystallographic data collection and refinement statistics

	T2-71 (PDB: 9ZV3)
Resolution range	43.49 - 3.24 (3.42 - 3.24)
Space group	*R 3 2 :H*
Unit cell	104.29, 104.29, 472.87; 90, 90, 120
Unique reflections	16281 (2302)
Multiplicity	5.7 (5.9)
Completeness (%)	100.00 (100.00)
Mean I/sigma(I)	7.1 (1.1)
R-merge	0.137 (1.420)
R-pim	0.069 (0.709)
$CC_{1/2}$	0.996 (0.457)
Reflections used in refinement	16274 (1314)
R-work	0.1910 (0.3262)
R-free	0.2356 (0.3728)
Number of non-hydrogen atoms	
macromolecules	5092
solvent	16
protein residues	600
RMS(bonds)	0.003
RMS(angles)	0.700
Ramachandran favored (%)	98.48
Ramachandran allowed (%)	1.52
Ramachandran outliers (%)	0.00
Average B-factor	
macromolecules	107
solvent	68
